# Factors influencing nutrition literacy in patients of type 2 diabetes mellitus: a cross-sectional study

**DOI:** 10.3389/fnut.2025.1654905

**Published:** 2025-12-18

**Authors:** Wenjuan Zhang, Yibao Zhang, Ziyu Sun, Jiaqi Wang, Yuhong Wu

**Affiliations:** 1 First Hospital of Shanxi Medical University, Taiyuan, China; 2 School of Nursing, Hangzhou Normal University, Hangzhou, China

**Keywords:** cross-sectional study, factors influencing, nutrition, nutrition literacy, type 2 diabetes mellitus

## Abstract

**Objective:**

To understand the current status of nutrition literacy in patients with type 2 diabetes mellitus and analyze its influencing factors, aiming to provide a basis for medical staff to construct nutrition management and education programs.

**Methods:**

A total of 790 patients with type 2 diabetes mellitus who met the inclusion exclusion criteria in Hangzhou City were selected from March 2024 to January 2025 using convenience sampling method. The researchers used a general demographic questionnaire and the Nutrition Literacy Scale for Patients with Type 2 Diabetes Mellitus.

**Results:**

Patients with type 2 diabetes mellitus scored (100.56 ± 22.27) on the Nutrition Literacy Scale. The results of multiple linear regression analysis showed that place of residence, education level, glycosylated hemoglobin, diabetic complications, comorbidities, and duration of the disease were the main factors affecting their nutrition literacy (*p* < 0.05).

**Conclusion:**

Nutrition literacy of patients with type 2 diabetes mellitus is at upper middle range and is affected by a variety of factors. Healthcare professionals should construct a nutrition management and education program based on the influencing factors to help patients improve their nutrition literacy level and form healthy eating behaviors.

## 1 Introduction

Type 2 Diabetes Mellitus (T2DM) is a chronic disease associated with insulin resistance and insufficient insulin secretion. According to the data released in the 10th edition of the International Diabetes Federation map (IDF) (1), the number of people with diabetes mellitus worldwide has climbed to 537 million by 2021, with China leading the world with 140 million diabetes mellitus people. IDF states that Type 2 diabetes mellitus is a classic nutrition-related disease and that nutrition therapy plays a central role in diabetes mellitus treatment. However, the latest study reported that the compliance rate of glycated hemoglobin, blood pressure and low density lipoprotein in adult T2DM patients in China is only 4.4%, which is much lower than the 21% in the United States, 15% in Canada, and 16% in Japan (2).

Nutrition literacy is the comprehensive ability of an individual to obtain and understand nutrition-related information and use it to make decisions that are conducive to healthy development (3). Studies have shown that inadequate nutrition literacy reduces an individual’s ability to make healthy dietary choices, leading to a greater preference for poorer-quality, high-fat diets, and that individuals with lower nutrition literacy seldom consult food labels and have greater difficulty in interpreting food labels and estimating appropriate food portions (4, 5). In addition, the policies of the World Health Organization’s Nutrition Vision and Action 2016–2025 (6) and China’s National Nutrition Program (2017–2030) (7) clearly indicate that vigorously promoting nutrition prevention and treatment of chronic diseases can effectively improve the health status of patients. Thus, it is imperative to assess the level of nutrition literacy in T2DM patients.

However, at present, the items of the existing assessment tools do not reflect the dietary characteristics of patients with T2DM and are mostly limited to functional nutrient literacy, lacking the assessment of nutrition information interaction and judging ability, which cannot comprehensively reflect the status of nutrient literacy in patients with T2DM. It poses a great limitation to the development and improvement of interventions (8). Therefore, this study used the nutrition literacy assessment tool for patients with type 2 diabetes mellitus, which was developed in the preliminary stage of this study, to comprehensively investigate their nutrition literacy status and the factors affecting it. This provides a basis for the development of a personalized nutrition intervention plan, so as to better support the health management of the patients.

## 2 Materials and methods

### 2.1 Study population and sampling criteria

Convenience sampling method was adopted, and T2DM in the endocrinology department and outpatient clinic of tertiary hospital in Hangzhou City were selected from March 2024 to January 2025 for the survey. Inclusion criteria: ① diagnosed with T2DM (9); ② age≥18 years old; ③ disease diagnosis for 6 months or more; ④ normal intelligence, no language communication barriers and voluntary participation in this study. Exclusion criteria: ① existing mental illness; ② physically weak and unable to complete the study. Using the Kendall estimation method (10), the sample size should be 5–10 times the number of independent variables, with a total of 18 independent variables at this stage, while increasing the questionnaire invalidity rate by 10%. The required sample size is 18 × 5 to 10/(1–10%) = 100 to 200 cases. To further refine the sample size, this study also used power analysis to determine the minimum sample size. This study ensures that, at a given significance level (*α* = 0.05), the expected association between the independent variable and the dependent variable (moderate effect size f^2^ = 0.15) can be detected. Typically, statistical power (1-*β*) must be no less than 0.8, and 10% invalid questionnaires must be taken into account. Using tools such as G*Power, the minimum required sample size was calculated to be 228 cases. This study was reviewed by the Ethics Committee of Hangzhou Normal University School of Nursing (No. 2023062).

### 2.2 Research instrument

① General information questionnaire: designed by the researcher, including gender, age, education level, marital status, place of residence, duration of illness, etc.

② Nutrition Literacy Scale for Patients with Type 2 Diabetes Mellitus: Based on the information-motivation-behavior theory and the hierarchical model of health literacy, a preliminary version of the nutrition literacy scale for patients with type 2 diabetes was developed. The final version of the scale was determined after expert consultation and large-sample reliability and validity testing (11). This scale consists of 4 dimensions and 31 items: nutrition attitudes, functional nutrition literacy, interactive nutrition literacy, and critical nutrition literacy. A 5-point Likert scale was used, with scores from 1 to 5 indicating “strongly disagree/never,” “disagree/occasionally,” “not sure/sometimes,” “Agree/Frequently,” and “Strongly Agree/Always.”Cronbach’s alpha coefficient was 0.946, scale content validity was 0.978, and calibration correlation validity with the Adult nutrition Literacy Scale was 0.518 ~ 0.760.

### 2.3 Data collection

The study was conducted in the form of a paper questionnaire, which was created by the researcher and checked by a highly qualified nurse with research experience. The researcher communicated with the head of the hospital to obtain consent to distribute the questionnaire together. When the patients came to the hospital for treatment, the survey subjects were selected according to the inclusion and exclusion criteria. After obtaining their informed consent, the paper version of the questionnaire was issued on the spot, and a unified guide was used to explain the purpose of the survey and the method of filling in the questionnaire. The questionnaire was asked to fill in the questionnaire truthfully within the stipulated period of time, and then recovered and checked after filling in the questionnaire.

### 2.4 Statistical analysis

It was analyzed using IBM SPSS Statistics version 26.0. General data of T2DM patients were expressed as frequency and percentage. Data that conform to a normal distribution in the total and dimension scores of nutrition literacy in patients with T2DM are expressed as mean and standard deviation, and in case of skewed distribution, median and inter quartile spacing are used (12). In the analysis of factors affecting nutrition literacy in patients with T2DM, *t*-tests and one-way ANOVA were used for data that satisfied normal distribution; conversely, non-parametric tests were used (13). Multifactorial analysis of nutrition literacy in T2DM patients was performed using multiple linear regression. *p* < 0.05 was used to indicate that the difference was statistically significant.

## 3 Results

### 3.1 General information

In this study, 800 questionnaires were actually distributed and 790 were effectively recovered, with an effective recovery rate of 98.75%. General information is shown in Table 1.

**Table 1:** General information of patients (*N* = 790).

Variable	Category	n(%)
Age (years)	<30	93 (11.8)
	30–59	560 (70.9)
	≥60	137 (17.3)
Gender	Male	481 (60.9)
	Female	309 (39.1)
Body mass index	Marasmus	102 (12.9)
	Normal	362 (45.8)
	Overweight	287 (36.3)
	Fat	39 (5.0)
Marital status	Married	539 (68.2)
	Unmarried	173 (21.9)
	Dissociaton	49 (6.2)
	Widowhood	29 (3.7)
Resident manner	Live with a spouse	289 (36.6)
	Live with your spouse and children	218 (27.6)
	Live with children	65 (8.2)
	Living alone	218 (27.6)
Residence	Rural	195 (24.7)
	Town	344 (43.5)
	City	251 (31.8)
Degree of education	Primary school and below	71 (9.0)
	Junior middle	151 (19.1)
	High school or junior	213 (27.0)
	College and above	355 (44.9)
Monthly household income (RMB)	<3,000	40 (5.1)
	3,000–7,000	219 (27.7)
	>7,000	531 (67.2)
Disease duration (years)	<1	168 (21.3)
	1–5	457 (57.8)
	6–10	98 (12.4)
	>10	67 (8.5)
Medical payment methods	Self-funded	198 (25.1)
	Medical insurance	592 (74.9)
Diabetic complications	Yes	177 (22.4)
	No	613 (77.6)
Other complications	Yes	579 (73.3)
	No	211 (26.7)
Glycated hemoglobin (%)	4.0 ~ 6.0	277 (35.1)
	6.1 ~ 8.0	398 (50.4)
	>8.0	115 (14.5)

Age is classified according to clinical and epidemiological groups, corresponding to young and younger populations, middle-aged populations, and elderly populations, respectively; Income levels are grouped according to the actual distribution of Chinese residents’ incomes, corresponding to low-income, middle-income, and high-income groups, respectively; The disease progression grouping primarily reflects the timeliness of intervention: corresponding to the newly diagnosed group, early disease progression group, mid-term disease progression group, and long-term disease progression group, respectively; Glycated hemoglobin is classified into groups according to clinical classification, corresponding to normal, impaired, and poor, respectively.

### 3.2 Nutrition literacy scores of patients with type 2 diabetes mellitus

In this study, the total score of nutrition literacy level of T2DM patients was (100.56 ± 22.27). The nutrition attitude dimension score was (12.71 ± 3.52), functional nutrition literacy dimension was (56.33 ± 12.93), interactive nutrition literacy dimension was (15.87 ± 4.76) and critical nutrition literacy dimension was (15.64 ± 4.91). Based on the mean scores of the items it can be seen that the scores of each dimension in descending order were functional nutrition literacy, nutrition attitudes, interactive nutrition literacy, and critical nutrition literacy, as shown in Table 2. The scores of the items are shown in Table 3.

**Table 2:** Scale scores and dimension scores.

Items	Scoring range	Score (mean ± standard deviation)	Item mean (mean ± standard deviation)
Total scale score	49 ~ 155	100.56 ± 22.27	3.24 ± 0.72
Nutrition attitude	4 ~ 20	12.71 ± 3.52	3.18 ± 0.88
Functional nutrition literacy	26 ~ 85	56.33 ± 12.93	3.31 ± 0.76
Interactive nutrition literacy	5 ~ 25	15.87 ± 4.76	3.17 ± 0.95
Critical nutritionl literacy	5 ~ 25	15.64 ± 4.91	3.13 ± 0.98

**Table 3:** Items with the lowest scores on each dimension of the scale.

Items	Mean ± SD
A3: I am willing to take the initiative to learn about diabetes nutrition	3.10 ± 1.25
B17: I know that I should pay attention to the nutrition labeling on the packaging when I shop for and eat food	2.95 ± 1.26
C3: I can understand the diabetes nutrition knowledge taught by professionals	2.95 ± 1.32
D5: I will synthesize nutrition information and my own eating habits and preferences to choose the right food	3.00 ± 1.29

A, Nutrition attitude; B, Functional nutrition literacy; C, Interactive nutrition literacy; D, Critical nutrition literacy.

### 3.3 Unifactorial analysis of nutrition literacy in patients with type 2 diabetes mellitus

The results of this study showed that place of residence, education level, diabetic complications, other complications, glycosylated hemoglobin, and duration of the disease were the factors influencing the level of nutrition literacy in patients with T2DM, and the differences were statistically significant (*p* < 0.05) (for details see Table 4).

**Table 4:** Results of unifactorial analysis.

Items	Score	Nutrition attitude	Functional nutrition literacy	Interactive nutrition literacy	Critical nutrition literacy
Age (years)					
<30	101.77 ± 22.24	12.90 ± 3.37	56.13 ± 13.23	16.87 ± 4.49	15.87 ± 4.89
30–59	100.57 ± 22.67	12.62 ± 3.66	56.50 ± 12.97	15.69 ± 4.83	15.76 ± 4.99
≥60	96.18 ± 19.54	12.82 ± 3.05	55.59 ± 12.25	13.88 ± 4.50	13.88 ± 4.26
*F*	0.415	0.139	0.047	2.957	1.194
*P*	0.661	0.874	0.954	0.054	0.305
Gender					
Male	100.06 ± 20.24	12.49 ± 3.39	56.10 ± 11.42	15.97 ± 4.65	15.71 ± 5.03
Female	101.39 ± 25.42	13.09 ± 3.74	56.71 ± 15.20	15.71 ± 4.95	15.50 ± 4.74
*t*	−0.421	−1.196	−0.336	0.383	−0.539
*P*	0.674	0.233	0.737	0.702	0.590
Body mass index					
Marasmus	99.74 ± 23.15	12.47 ± 3.86	57.11 ± 13.47	15.00 ± 5.41	15.16 ± 5.18
Normal	102.97 ± 22.05	13.15 ± 3.47	57.05 ± 12.73	16.50 ± 4.77	16.27 ± 4.90
Overweight	96.45 ± 22.01	11.95 ± 3.47	55.02 ± 12.97	15.03 ± 4.49	14.45 ± 4.82
Fat	100.5 ± 24.21	13.00 ± 3.56	54.9 ± 15.21	15.70 ± 4.60	16.90 ± 4.36
*F*	1.224	1.700	0.411	1.602	2.249
*P*	0.302	0.168	0.746	0.190	0.084
Marital status					
Married	98.68 ± 21.87	12.42 ± 3.56	55.80 ± 12.88	15.37 ± 4.73	15.09 ± 4.82
Unmarried	106.43 ± 23.22	13.52 ± 3.42	58.27 ± 13.46	17.48 ± 4.58	17.16 ± 5.00
Dissociaton	91.20 ± 17.66	12.80 ± 3.42	50.40 ± 8.91	12.60 ± 3.91	15.40 ± 5.08
Widowhood	101.50 ± 6.36	12.50 ± 2.12	56.50 ± 4.95	17.00 ± 2.83	15.50 ± 2.12
*F*	1.980	1.338	0.852	3.655	2.483
*P*	0.118	0.263	0.467	**0.013**	0.062
Resident manner					
Live with a spouse	104.52 ± 22.37	13.18 ± 3.26	57.45 ± 12.62	16.98 ± 4.42	16.90 ± 4.99
Live with your spouse and children	100.52 ± 20.14	12.69 ± 3.50	57.38 ± 11.83	15.70 ± 4.64	14.74 ± 4.78
Live with children	104.50 ± 28.87	13.50 ± 3.79	59.50 ± 15.42	16.75 ± 5.32	14.75 ± 4.50
Living alone	96.09 ± 24.78	12.20 ± 3.82	53.13 ± 14.58	14.91 ± 5.13	15.86 ± 4.89
*F*	1.434	0.823	1.598	1.966	2.486
*P*	0.234	0.483	0.191	0.120	0.062
Residence					
Rural	94.14 ± 19.79	11.82 ± 3.31	53.00 ± 11.57	14.73 ± 4.81	14.59 ± 4.60
Town	105.82 ± 19.85	13.60 ± 3.23	58.91 ± 12.23	16.82 ± 4.38	16.49 ± 4.44
City	107.38 ± 24.88	13.56 ± 3.74	59.97 ± 14.27	17.08 ± 4.54	16.77 ± 5.41
*F*	9.284	7.040	7.321	6.258	4.920
*P*	*<* **0.001**	*<* **0.001**	*<* **0.001**	**0.002**	**0.008**
Degree of education					
Primary school and below	85.21 ± 24.61	10.88 ± 3.90	47.74 ± 15.39	13.56 ± 4.60	13.03 ± 4.39
Junior middle	93.06 ± 18.47	11.65 ± 2.87	52.10 ± 10.62	14.42 ± 4.63	14.90 ± 5.23
High school or junior	99.25 ± 12.88	12.33 ± 2.98	55.23 ± 8.06	15.96 ± 4.17	15.73 ± 3.69
College and above	112.43 ± 22.24	14.39 ± 3.38	63.23 ± 12.30	17.68 ± 4.62	17.14 ± 5.14
*F*	18.487	12.494	17.841	8.908	6.518
*P*	*<* **0.001**	*<* **0.001**	*<* **0.001**	*<* **0.001**	*<* **0.001**
Monthly household income (RMB)					
*<*3,000	103.07 ± 18.70	13.21 ± 3.09	57.86 ± 11.06	17.14 ± 4.52	14.86 ± 4.40
3,000–7,000	99.30 ± 24.27	12.30 ± 3.74	55.79 ± 13.35	15.13 ± 5.72	16.09 ± 5.39
>7,000	100.80 ± 21.88	12.83 ± 3.49	56.39 ± 13.01	16.04 ± 4.34	15.54 ± 4.78
*F*	0.185	0.592	0.148	1.285	0.438
*P*	0.831	0.554	0.863	0.279	0.646
Disease duration (years)					
*<*1	96.60 ± 22.30	12.49 ± 3.53	54.31 ± 13.18	14.49 ± 4.66	15.31 ± 4.86
1–5	103.39 ± 21.86	12.93 ± 3.49	57.46 ± 12.82	16.80 ± 4.65	16.20 ± 4.85
6–10	93.70 ± 24.77	11.8 ± 3.85	54.70 ± 14.04	13.65 ± 4.48	13.55 ± 5.19
>10	95.05 ± 20.41	12.52 ± 3.47	53.81 ± 12.15	14.19 ± 4.42	14.52 ± 4.75
*F*	2.211	0.695	1.004	5.403	2.245
*P*	0.088	0.556	0.392	*<* **0.001**	0.084
Medical payment methods					
Self-funded	100.39 ± 22.81	12.98 ± 3.56	55.56 ± 12.83	15.85 ± 4.94	16.00 ± 4.57
Medical insurance	100.62 ± 22.12	12.61 ± 3.52	56.63 ± 13.00	15.88 ± 4.70	15.50 ± 5.05
*t*	−0.067	0.703	−0.549	−0.041	0.675
*P*	0.946	0.483	0.584	0.967	0.500
Diabetic complications					
Yes	93.69 ± 23.08	11.38 ± 3.89	53.38 ± 13.93	14.13 ± 4.89	14.81 ± 5.50
No	102.54 ± 21.70	13.10 ± 3.32	57.18 ± 12.54	16.38 ± 4.62	15.88 ± 4.72
*t*	2.455	3.048	1.805	2.944	1.327
*P*	**0.015**	**0.003**	0.072	**0.004**	0.186
Other complications					
Yes	91.21 ± 17.70	11.58 ± 3.27	51.36 ± 10.56	14.30 ± 4.40	13.98 ± 4.53
No	113.19 ± 21.64	14.25 ± 3.28	63.04 ± 12.86	18.00 ± 4.40	17.89 ± 4.53
*t*	8.163	5.912	7.292	6.081	6.256
*P*	*<* **0.001**	*<* **0.001**	*<* **0.001**	*<* **0.001**	*<* **0.001**
Glycated hemoglobin (%)					
4.0 ~ 6.0	105.32 ± 22.90	13.50 ± 3.69	58.80 ± 13.06	16.58 ± 4.98	16.44 ± 5.07
6.1 ~ 8.0	97.45 ± 23.48	12.01 ± 3.11	55.64 ± 15.02	15.18 ± 4.76	15.64 ± 5.39
>8.0	94.18 ± 19.27	11.00 ± 3.52	52.75 ± 11.30	15.00 ± 4.35	14.42 ± 4.30
*F*	6.275	7.41	5.265	2.833	3.983
*P*	**0.002**	*<* **0.001**	**0.006**	0.061	**0.020**

The bolded part represents that the variable has statistical significance in different populations.

### 3.4 Multivariate analysis of nutrition literacy in type 2 diabetes patients mellitus

In this study, we constructed a multiple linear regression model to analyze the total nutrition literacy score and the scores of each dimension of T2DM patients as dependent variables. The variables that reached a statistically significant level in the univariate analysis as independent variables. The results showed that patients’ place of residence, education level, glycosylated hemoglobin, diabetic complications, other complications, and disease duration were the main influencing factors (*p* < 0.05), as shown in Table 5.

**Table 5:** Results of multivariate linear regression.

Items	*B*-value	SE	*Β*-value	*t*	*P*	VIF
(a) Nutrition literacy score						
(Constant)	88.203	4.123		21.391	*<*0.001	
Residence						
Rural (Ref)						
City	6.452	2.758	0.133	2.339	**0.020**	1.199
Degree of education						
Primary school and below (ref)						
Junior middle	8.582	3.862	0.161	2.222	**0.027**	1.951
High school or junior	15.403	3.876	0.297	3.974	*<* **0.001**	2.079
College and above	21.734	3.568	0.473	6.092	*<* **0.001**	2.241
Glycosylated hemoglobin						
4.0% ~ 6.0% (ref)						
>8.0%	−1.447	4.168	−0.02	−0.347	**0.022**	1.205
Other complications						
No						
Yes	−19.462	2.510	−0.433	−7.752	*<* **0.001**	1.159
Diabetic complications						
No						
Yes	−5.240	2.838	−0.098	−1.847	0.066	1.054
(b) Nutrition attitude						
(Constant)	10.950	0.717		15.280	*<*0.001	
Degree of education						
Primary school and below (ref)						
High school or junior	1.394	0.674	0.170	2.069	**0.040**	2.079
College and above	2.718	0.620	0.374	4.383	*<* **0.001**	2.241
Glycosylated hemoglobin						
4.0% ~ 6.0% (ref)						
>8.0%	−1.533	0.724	−0.132	−2.116	**0.036**	1.205
Other complications						
No						
Yes	−2.308	0.436	−0.325	−5.290	*<* **0.001**	1.159
Diabetic complications						
No						
Yes	−1.144	0.493	−0.136	−2.319	**0.021**	1.054
(c) Functional nutrition literacy						
(Constant)	50.944	2.265		22.497	*<*0.001	
Residence						
Rural (ref)						
City	3.342	1.688	0.119	1.980	**0.049**	1.199
Degree of education						
Primary school and below (Ref)						
Junior middle	4.938	2.358	0.160	2.094	**0.037**	1.943
High school or junior	8.731	2.350	0.290	3.715	*<* **0.001**	2.040
College and above	13.059	2.166	0.490	6.029	*<* **0.001**	2.206
Glycosylated hemoglobin						
4.0% ~ 6.0% (ref)						
>8.0%	−0.196	2.534	−0.005	−0.077	**0.040**	1.189
Other complications						
No						
Yes	−10.093	1.536	−0.387	−6.569	*<* **0.001**	1.159
(d) Interactive nutrition literacy						
(Constant)	14.317	1.293		11.071	*<*0.001	
Residence						
Rural (ref)						
City	1.348	0.639	0.130	2.110	**0.036**	1.266
Degree of education						
Primary school and below (ref)						
High school or junior	2.127	0.904	0.192	2.351	**0.020**	2.227
College and above	2.698	0.842	0.275	3.203	**0.002**	2.457
Disease duration (years)						
*<*1 (ref)						
1 ~ 5	2.299	0.749	0.232	3.072	**0.002**	1.899
Other complications						
No						
Yes	−3.951	0.596	−0.412	−6.633	*<* **0.001**	1.283
(e) Critical nutrition literacy						
(Constant)	14.154	0.950		14.896	*<*0.001	
Degree of education						
Primary school and below (ref)						
Junior middle	2.130	0.989	0.181	2.152	**0.033**	1.943
High school or junior	3.213	0.986	0.281	3.259	*<* **0.001**	2.040
College and above	3.253	0.909	0.321	3.579	*<* **0.001**	2.206
Other complications						
No						
Yes	−3.696	0.645	−0.373	−5.734	***<*0.001**	1.159

(a) Total score of nutritional literacy: *R*^2^ = 0.450, Adjusted *R*^2^ = 0.426, *F* = 18.577, *P <* 0.001; (b) Nutrition attitude: *R*^2^ = 0.337, Adjusted *R*^2^ = 0.308, *F* = 11.535, *P <* 0.001; (c) Functional nutrition literacy: *R*^2^ = 0.387, Adjusted *R*^2^ = 0.363, *F* = 16.158, *P <* 0.001; (d) Interactive nutrition literacy: *R*^2^ = 0.418, Adjusted *R*^2^ = 0.361, *F* = 9.330, *P <* 0.001; (e) Critical nutrition literacy: *R*^2^ = 0.252, Adjusted *R*^2^ = 0.223, *F* = 8.639, *P <* 0.001. Ref is the abbreviation for Reference. The bolded part represents that the variable has statistical significance in different populations.

## 4 Discussion

### 4.1 Current status of nutrition literacy in type 2 diabetes mellitus

Nutrition literacy score of T2DM patients in this study was (100.56 ± 22.27) and mean of items was (3.24 ± 0.72). It was in the upper middle range compared to the median score (77.5) of the scale used in this study, which is consistent with previous studies (8, 14). Another study showed that the nutrition literacy of patients with diabetic foot was at a low level (15), the reason for which may be related to the inconsistency of the study population and the insufficient knowledge base and behavioral skills possessed by the patients who developed complications themselves (15).

The items in the function literacy dimension had the highest mean scores. But there is still room for improvement, item B17 “I know that I should pay attention to the nutrition labeling on the packaging when I shop for and eat food” scoring the lowest, which is in line with similar studies (16). Nutrition labeling, as an important source of information on food packages, which can help patients make healthier dietary choices. However, patients’ attention to nutrition labeling is currently weak, and healthcare professionals should enhance patient education on food choices and nutrition label interpretation.

Item A3“I am willing to take the initiative to learn about diabetes nutrition” scored the lowest on the nutrition attitude dimension. The reason for this may be that patients continue to accumulate experience and knowledge during medical treatment, but the long period of self-management makes patients bored and slack. Therefore, the strategy of “teaching for fun” (17) can be adopted, in which nutrition information is interspersed with recreational activities, such as soap operas, songs, games and so on. Some studies have indicated (18) that the use of virtual technology in health education to enable patients to pre-experience the symptoms of complications can effectively enhance patients’ motivation to learn.

Meanwhile, item C3 “I can understand the diabetes nutrition knowledge taught by professionals” scored the lowest among the interactive nutrition literacy dimensions in this study, which may be due to the overly theoretical teaching of healthcare professionals (19). Healthcare professionals need to be more attuned to the actual needs and comprehension ability of the patients.

Critical nutrition literacy items had the lowest mean scores, suggesting that patients’ overall level of nutrition literacy may be influenced by this dimension. Healthcare professionals can refer to a series of catechisms jointly constructed by experts from several European countries, focusing on the development of citizens’ ability to acquire, analyze and evaluate information (20). In addition, item D5“I will synthesize nutrition information and my own eating habits and preferences to choose the right food,” which may be related to the fact that patients rely too much on the advice of doctors or nutrition specialists and neglect the importance of self-learning and thinking.

### 4.2 Factors affecting nutrition literacy among patients with type 2 diabetes mellitus

In this study, we used multiple linear regression analysis to explore the factors influencing nutrition literacy in type 2 diabetes mellitus patients and systematically tested the modeling assumptions and potential confounders. First, the residuals were confirmed to be approximately normally distributed by residual Q-Q plots and Shapiro–Wilk tests, and natural logarithmic transformations were used for variables that mildly deviated from normality in order to improve the model fit. Second, the Breusch-Pagan test and the scatterplot of residuals-fitted values show that the model satisfies the assumption of homoskedasticity, and if subsequent analyses reveal localized heteroskedasticity, the use of robust standard errors may be considered for correction. Finally, the multicollinearity diagnostics showed that the variance inflation factors (VIF) of all independent variables were <10, indicating no serious covariance problems among the predictor variables (21). In this study, possible confounders were excluded through stepwise regression and sensitivity analysis (re-fitting the model after removing extreme values) to ensure the robustness of the results, and the final model-adjusted R^2^ was 0.45, indicating that the selected variables explained the variation in nutrition literacy to a good extent.

Patients living in urban areas had higher levels of nutrition literacy than those in rural areas, and significantly higher levels of functional and interactive nutrition literacy dimensions than those in rural areas. This may be due to the fact that urban patients have higher levels of literacy, better social support, more opportunities to attend health lectures and engage with professionals (22). Chowdhury’s study showed that rural patients generally have a misunderstanding of knowledge related to diabetes nutrition and limited sources of specialized information (23). Therefore, there is a need to reduce the urban–rural gap by popularizing professional nutrition knowledge to rural patients through more channels and methods.

Degree of education is one of the important factors affecting the level of nutrition literacy in patients with T2DM, and patients with a college level and above scored significantly higher in the total nutrition literacy score and in the four dimensions than those with an elementary school level and below, which is in line with the relevant findings (24). Studies have shown that highly educated people use health apps to gain nutrition knowledge 2.8 times more than those with low education (25). In the digital age, possessing sound digital health literacy enables one to efficiently sift through vast amounts of online information and discern scientifically sound nutritional knowledge. This further demonstrates that individuals with robust digital health literacy can directly enhance their nutritional literacy, thereby more effectively improving their health (26). In addition, highly educated patients are more aware and capable of self-learning and thinking, and will take the initiative to acquire and update their nutrition knowledge (27). Therefore, for patients with a low level of education, healthcare institutions should optimize and improve nutrition education materials and methods, using more vivid examples and graphic metaphors as much as possible to help patients understand complex nutrition concepts.

Overall nutrition literacy, nutrition attitude and functional nutrition literacy scores of patients with higher glycated hemoglobin were significantly lower than those of patients with normal glycated hemoglobin. Meanwhile, Rivera found that diabetic patients with lower levels of nutrition literacy had significantly higher mean glycosylated hemoglobin (8.6%) than those with higher levels of nutrition literacy (6.9%) (28). It can be seen that the influence of nutrition literacy and glycated hemoglobin may be bidirectional. On the one hand, patients with low levels of nutrition literacy may lack knowledge about how to rationalize their diets and control their sugar intake (8). On the other hand, patients with higher glycated hemoglobin may lack sufficient understanding and attention to the treatment and management of diabetes (29). Therefore, for patients with higher glycated hemoglobin, healthcare providers should equally focus on raising patients’ awareness of nutrition therapy in addition to strengthening nutrition education. Future large-sample longitudinal studies are needed to confirm the relationship between the two.

The management of chronic non-communicable diseases is a global research priority, and the prevalence of chronic diseases in China is as high as 55–98% (30, 31), which seriously affects patients’ quality of life and psychological status. The results of this study showed that T2DM patients with comorbidities tended to exhibit lower levels of nutrition literacy in terms of nutrition attitudes, functional nutrition literacy, interactive nutrition literacy, and critical nutrition literacy. Analyzing the reasons for this, on the one hand, it may be that comorbidities cause patients to face more health challenges, making it difficult for them to focus on learning and practicing nutrition literacy. On the other hand, it may be related to the fact that such patients often transition between care settings. The treatment of comorbidities requires specific dietary restrictions or modifications, which increases the complexity of dietary management and makes it difficult to integrate and utilize the nutrition information that patients receive. Previous studies have shown (32) that care coordination reduces readmission rates and mortality in patients with multiple chronic diseases. Close communication between healthcare professionals from different disciplines is an important factor in facilitating care coordination, so healthcare professionals should jointly develop multidisciplinary and multimorbid nutritional therapy programs. In addition, the need to train senior technicians in clinical nutrition nursing has been clarified in the Healthy China 2030 Planning Outline (33) and the National Nutrition Program (2017–2030) (7). Therefore, the relevant authorities should strengthen the training of nutrition specialist nurses, and further assess whether the existing core competencies of nutrition specialist nurses can meet the nutrition needs of multimorbid patients.

Patients with complications had lower scores on the nutrition attitude dimension compared to the general population, which is consistent with the results of Bukari’s study (34). It is possible that the complications may have led to the patients’ limited access to and preparation of food, further weakening their confidence in nutrition therapy (35). A Meta-integration similarly pointed out that patients with diabetic retinopathy tend to choose convenient food nearby, gradually ignoring the importance of dietary management (36). Therefore, healthcare professionals can customize education programs from the introduction of successful cases, and also actively carry out family education for patients with limited vision or mobility.

Disease duration is a factor influencing interactive nutrition literacy in T2DM patients, but it is not the case that the longer the disease duration, the higher the level of interactive nutrition literacy. The results of this study showed that patients with a disease duration of 1–5 years had the highest scores, probably due to the fact that patients at this stage have higher motivation and willingness to learn about disease management (37). Patients with a disease duration of less than 1 year may be in a period of adaptation to the disease because they have just been diagnosed with diabetes. For patients with a disease duration of more than 5 years, long-term disease management has led to a certain degree of psychological fatigue or slackness. Therefore, for patients with different disease duration, the focus of healthcare professionals’ education strategies should be different (38).

There are also some limitations of this study. Due to limited time and human resources, this study was limited to type 2 diabetes patients in Hangzhou, which is a small and unrepresentative sample size. In the future, this scale should be applied to hospitals in different regions and grades, and the scale should be validated and improved through a large sample survey. This study has not yet analyzed the relationship between nutrition literacy and other important variables (e.g., dietary adherence), especially in the interactive and critical dimensions of nutrition information. Nowadays, in the context of the Internet era, access to and identification of nutrition information is particularly important for disease management. In addition, the bidirectional relationship between nutrition literacy and glycosylated hemoglobin mentioned in this study cannot be inferred as a causal relationship due to the cross-sectional design and needs to be further tested. Future research may combine cross-sectional and longitudinal study designs to both track dynamic trends and reveal causal relationships. Integrating quantitative and qualitative methods, such as interviews or focus groups, will provide a deeper understanding of respondents’ motivations, perspectives, and behaviors. Finally, although we adjusted for basic demographic characteristics such as age, gender, and education level in the regression analyses, we still need to consider possible residual confounding from unmeasured psychosocial factors (e.g., health beliefs, self-efficacy), and future research could further explore the complex pathway relationships in conjunction with structural equation modeling.

## 5 Conclusion

This study shows that nutrition literacy of type 2 diabetes patients is at upper middle range, which should be further improved, especially the cultivation of patients’ ability to interact with and judge nutrition information. Factors influencing the level of nutrition literacy of type 2 diabetes patients include patients’ place of residence, literacy level, glycosylated hemoglobin, diabetic complications, comorbidities, and duration of the disease. Therefore, healthcare professionals can give individualized and precise guidance on nutritional health interventions for patients’ specific conditions.

## Data Availability

The original contributions presented in the study are included in the article/supplementary material, further inquiries can be directed to the corresponding author.
